# The m-TORC1 inhibitor Sirolimus increases the effectiveness of Photodynamic therapy in the treatment of cutaneous Squamous Cell Carcinoma, impairing NRF2 antioxidant signaling

**DOI:** 10.7150/ijbs.94883

**Published:** 2024-08-06

**Authors:** Jimena Nicolás-Morala, Marta Mascaraque-Checa, María Gallego-Rentero, Andrea Barahona, Edgar Abarca-Lachen, Elisa Carrasco, Yolanda Gilaberte, Salvador González, Ángeles Juarranz

**Affiliations:** 1 Department of Biology, Universidad Autónoma de Madrid, Madrid, Spain.; 2 Department of Experimental Dermatology and Skin Biology, Instituto Ramón y Cajal de Investigación Sanitaria, IRYCIS, 28034 Madrid, Spain.; 3 Faculty of Health Sciences, Universidad San Jorge, 50830 Villanueva de Gállego, Spain.; 4 Centro de Biología Molecular Severo Ochoa (CBM), Instituto Universitario de Biología Molecular-IUBM (Universidad Autónoma de Madrid), Madrid, Spain.; 5 Dermatology service, Hospital Miguel Servet, Zaragoza (50009), Spain.; 6 Department of Medicine and Medical Specialties, Universidad Alcalá de Henares, 28805 Madrid, Spain.

**Keywords:** Cutaneous Squamous Cell Carcinoma, Photodynamic Therapy, Rapamycin, Tumor Resistance, Antioxidant Response

## Abstract

Squamous Cell Carcinoma (SCC) is a subtype of Non-Melanoma Skin Cancer, the most common group of malignancies worldwide. Photodynamic therapy (PDT) is a non-invasive treatment approved for specific subtypes of SCC. Some malignancies resist PDT, forming more aggressive tumors and multiple relapses. Thus, new approaches aimed at optimizing the response to PDT are needed. The mTORC1 inhibitor rapamycin, also known as Sirolimus (SRL), interferes with protein synthesis and cell metabolism. The use of SRL as an immunosuppressant is associated to lower rates of SCC in kidney-transplanted patients, which are frequently affected by this pathology. We have evaluated SRL pre-treatment efficacy to enhance the damage induced by PDT with Methyl 5-aminolevulinate in two different cutaneous SCC established cell lines (SCC13 and A431) *in vitro* and therapy sensitization in PDT-resistant cell lines. We tested for the first time the SRL + PDT combination in a SKH-1 mouse model of photocarcinogenesis, diminishing the frequency of lesions and restraining tumor growth. Molecular studies revealed that protoporphyrin IX and reactive oxygen species production induced by PDT were promoted by SRL pre-treatment. Lastly, SRL modifies the expression and intracellular location of NRF2, interfering with the downstream antioxidant response modulated by NQO1 and HO-1. In conclusion, we propose SRL as a potential adjuvant to enhance PDT efficacy for SCC treatment.

## Introduction

Non-Melanoma Skin Cancer (NMSC) is originated in epidermal keratinocytes and it represents the most common type of cancer worldwide. NMSC preferentially affects white-skinned populations with I and II skin phototypes 1. The two main subtypes of NMSC are Basal Cell Carcinoma (BCC) and Squamous Cell Carcinoma (SCC). BCC is more prevalent but less aggressive than SCC. However, immunosuppressed patients are more frequently affected by SCC than BCC, displaying a more aggressive behaviour 2. Although SCC is less prevalent in the general population, this malignancy occupies the 4^th^ place on new cancer cases worldwide, according to Global Cancer Observatory data (2020) 3.

Surgery is the optimal standard treatment for NMSC, with 97.5% clearance at 2-year follow-up 4. However, surgery is invasive and involves elevated health care costs. Therefore, other non-invasive therapies with lower costs and good cosmetic results are under development, especially for superficial forms. Examples of them are radiotherapy, intratumoral methotrexate, topical chemotherapy (5-fluorouracil, diclofenac, Imiquimod) and photodynamic therapy (PDT) 5.

PDT is currently approved as a non-invasive therapy by the Food and Drug Administration (FDA) and European Medicines Agency (EMA) for the treatment of Actinic Keratosis (AK, a premalignant lesion of SCC), and some specific subtypes of SCC and BCC. This therapy has gained great relevance due to its optimal cosmetic results, high efficacy rates (89.5% clearance), and lower health care-related costs. PDT constitutes an excellent therapeutic option for candidates who reject surgery 6, 7.

PDT is based on the combination of three elements: light, oxygen, and a photosensitizer (PS) 8. PDT action mechanism relies on specific PS accumulation within tumor cells due to their metabolic alterations 9, 10. The exposition of the tumoral area to light of adequate wavelength (red light is preferred due to its higher tissue penetration) leads to the formation of reactive oxygen species (ROS), mainly singlet oxygen (^1^O_2_), triggering tumor cell death 11. Among the approved molecules for NMSC photodynamic treatment are protoporphyrin IX (PpIX) precursors, such as δ-aminolevulinic acid (ALA) and its methyl-ester, methyl 5-aminolevulinate (MAL) 12.

Tumors are complex systems in which malignant cells coexist and interact among them and with other components of the tumor microenvironment, such as cancer associated fibroblasts (CAFs), immune cells, the extracellular matrix and the vascularization system. All these interactions can modulate tumor development and evolution 13. In certain cases, tumor cells can develop resistance to different therapies. Although PDT constitutes a highly recommended option for NMSC treatment, the resistance phenomenon has also been described after this therapy.

In addition, either using *in vitro* approaches or in patients, it was observed that malignant cells surviving to several cycles of PDT not only become resistant, but they also display higher aggressiveness than their original counterparts 14, 15, 16. There have been different mechanisms by which malignant cells become resistant. Some of them are (i) modifications in the enzymes of the heme synthesis pathway leading to reduced accumulation of PpIX; (ii) drug pumps P-glycoprotein: ABCG1 and ABCG2 enhanced activity; (iii) altered expression of apoptotic proteins or (iv) cellular antioxidant defence mechanisms 15, 17.

In this scenario, strategies to target the activation of molecular pathways that mediate antioxidant responses are gaining great relevance to enhance the efficacy of PDT. Several proteins as Superoxide dismutase (SOD), NAD(P)H:quinone oxidoreductase 1 (NQO1) and Heme-Oxygenase 1 (HO-1) increase their levels after PDT in order to confront ROS upon irradiation 18. The master regulator controlling the expression of these proteins is a specific transcription factor called nuclear factor-erythroid 2 related factor 2 (NRF2) 19. NRF2 is activated under stress conditions, including ROS and ultraviolet radiation (UVR). In basal conditions, NRF2 is ubiquitinated and subsequently degraded. However, under stress conditions, NRF2 undergoes nuclear translocation and binds Antioxidant Response Elements (AREs) promoting the expression of antioxidant response target genes 20, 21.

The use of neo-adjuvant and adjuvant therapies (before and after PDT, respectively) is a common practice to optimize PDT efficacy and improve patient quality of life. Some of the components previously used for PDT optimization are chemotherapeutic agents such as cisplatin and 5-fluorouracil, methotrexate, vitamin D and metformin 17, 22, 23, 24, 25. In this context, we propose Rapamycin as a candidate compound for pre-treatment prior to PDT in SCC.

The mTORC1 inhibitor Sirolimus (SRL), also known as rapamycin, is obtained from the bacteria *Streptomyces hygroscopicus*. Since this compound interferes with protein synthesis and cell metabolism, it can also affect the activity of immune cells 26. SRL is approved for clinical use as an immunosuppressant (Rapamune - EMEA/H/C/000273 - IB/0189). It was reported that kidney transplant patients treated with SRL to prevent rejection presented lower SCC incidence, a very common malignancy among these patients 27, 28, 29. Furthermore, different studies have shown SRL suitability as an adjuvant therapy for different cancer treatment strategies 30. SRL applied in combination with chemotherapy has been highly beneficial in colon cancer and it inhibits liver and lung metastases in mouse models 31. In addition, SRL combined with PDT produces increased damage to *in vitro* immortalized endothelial cells of the tumor vasculature 32. Furthermore, SRL enhanced chlorine e6-PDT and photothermal therapy efficacy for metastatic breast cancer treatment in mouse models 33. However, no evidence has been found regarding the effects of SRL before PDT for cutaneous SCC.

Considering this gap in knowledge, we have studied the efficacy of SRL pre-treatment to enhance the damage induced by MAL-PDT in SCC models. To evaluate the response to SRL+PDT of SCC cells, we have used two different cutaneous SCC established cell lines, cultured *in vitro* in two-dimensional and three-dimensional models (spheroids), as well as their corresponding PDT-generated resistant cell lines 25, 34. In light of the satisfactory results obtained *in vitro*, we moved onto an *in vivo* system to test, for the first time, the effect of the combination of SRL+PDT in a mouse model of photocarcinogenesis, generating promising results.

To uncover the subjacent mechanism, we studied the levels of PpIX and ROS and found a rise in both following SRL pre-treatment prior to PDT. Finally, we identified a modulation of the antioxidant response caused by SRL-induced mTOR inhibition. Subsequently, SRL is proposed as an excellent agent to promote the effectiveness of PDT for SCC treatment.

## Materials and methods

### Cell lines

We have employed two cell lines of cutaneous SCC, SCC13 cell line obtained from a facial SCC of a 56-year-old female 35 and kindly provided by Dr Miguel Quintanilla (Biomedical Sciences Institute, Alberto Sols, Madrid, Spain), and A431 obtained from an 85-year-old female patient with epidermoid carcinoma 36, purchased at ATCC (American Type Culture Collection).

Cells were grown in a CO_2_ incubator at 37⁰C, 95% humidity and 5% CO_2_ (*Hera Cell*). Cells were cultured within 25 cm^2^ flask (Fisher Scientific, Waltham, Massachusetts, USA) in Dulbecco's Modified Eagle's Medium (DMEM) supplemented with 1 % Hyclone Penicillin-Streptomycin 100X (Penicillin: 10000 U/ml; Streptomycin: 10000 μg/ml in 0.85 % NaCl) solution and 10% Fetal Bovine Serum (FBS). For amplification, cells were trypsinized with 1 mM EDTA/0.25% Trypsin (Fisher Scientific, Waltham, Massachusetts, USA). For two-dimensional cultures, cells were seeded in multi-well plates with 6, 12, 24, or 48 wells (Fisher Scientific, Waltham, Massachusetts, USA) and treated when cultures reached 20-30% confluence.

In addition, the cell populations resistant to PDT used in this study were previously generated in the laboratory by subjecting the original cultures (parental cells) to 10 cycles of ascendant doses of PDT according to the protocol published to generate SCC13 10G 34 and A431 10G 25. Briefly, cells were grown in F25 flasks, incubated 5 h with MAL and irradiated to complete one PDT cycle. Each cycle of PDT induced around 80-90% of cell lethality. Surviving cells were harvested 24 h after each PDT application and reseeded until they were fully recovered, reaching 100% confluence. Once recovered, cells were subjected to the next PDT cycle and so forth. The MAL concentration employed to develop PDT-resistant cells was 1 mM MAL, and the irradiation doses employed are indicated in **Table 1**.

### Spheroids formation

The formation of spheroids was performed following the protocol published by Adhikary *et al.* 37 in 2013. Briefly, cells were seeded in six-well plates previously coated with 1.2% poly-HEMA (2-hydroxyethyl methacrylate, Sigma, Darmstadt, Deutschland) in ethanol 96% that has been evaporated overnight and then sterilized irradiating the plates under UV light for 1 h. The cells then were seeded on 6-well plates at a concentration of 20,000 cells/mL and cultured in specific medium containing DMEM: F12 (1:1) and supplemented with 0.4% BSA (Sigma, Darmstadt, Deutschland), 20 ng/mL of EGF (Sigma, Darmstadt, Deutschland), 4 μg/mL insulin (Sigma, Darmstadt, Deutschland) and 2% of B27 supplement (Invitrogen, Waltham, Massachusetts, USA). The spheroids were grown for 5 days to reach 100 µm diameter in size before performing the treatments.

### Treatments

For 2D assays on multi-well plates, cells were seeded at an approximate concentration of 25,000 cells/mL. Once cells reached the desired confluence (20-30%), SRL (SelleckChem Houston, Texas, United States) was added from a 2.5 mM stock in DMSO into the complete medium (DMEM + 10% FBS) to reach different concentrations (between 1 and 100 nM) and maintained for 48 h. The SRL concentration selected for molecular studies was 50 nM, based on previous *in vitro* and *in vivo* studies 32, 38, 39 and on the results of our experiments using combined treatments.

To apply PDT, the plates were incubated with 0.5 mM MAL in DMEM without FBS for 5 h followed by red light irradiation at different fluences (0.6 J/cm^2^ - 12 J/cm^2^). The concentration of MAL used was selected based on previous works carried out by our group. This concentration does not induce cell toxicity by itself, but it is able to provoke high cell photosensitivity as a function of the light dose applied 41, 42. After irradiation, cultures were washed with complete medium and incubated under standard conditions until evaluation.

For the combination of SRL + PDT, after the 48 h period of exposition to SRL in the culture media, cells were washed with Phosphate Buffered Saline (PBS) and immediately subjected to PDT (MAL + red light).

### Evaluation of cell viability

Cell viability in two-dimensional cultures was measured using the MTT (3-(4,5-dimethylthiazol-2-yl)-2,5-diphenylthiazole bromide (Sigma, Darmstadt, Deutschland) assay 25. Briefly, 24 h after the treatments cells were incubated for 3 h with 100 μg/mL of MTT diluted in DMEM. The formazan crystals generated were solubilized with dimethyl sulfoxide (DMSO) and absorbance at 542 nm wavelength was determined by spectrophotometry using a Tecan SPECTRAFluor Plus Microplate Reader.

For the evaluation of synergetic, additive or antagonistic effect, calculations on cell viability were performed as follows: DL = (log cell survival percentage SLR + log cell survival percentage PDT) - log cell survival percentage combination.

The mathematical interpretation states that a value significantly lower than 2 means there is an antagonistic effect. A value similar to 2 reveals that the effects of both therapies are additive and a value higher than 2 indicates the presence of a synergistic effect 40.

In the case of spheroids, cell viability was assessed using Acridine Orange/Propidium Iodide (AO/PI) (Fisher Scientific, Waltham, Massachusetts, USA) staining. To this end, 50 μg/mL of each fluorochrome were added directly to the wells. The emission was captured using an inverted fluorescence microscope under blue (λ = 450-490 nm) and green (λ = 590 nm) light excitation for AO and PI, respectively. All cells (dead and alive) emit green fluorescence owing to AO staining, which allows the determination of the total area of the spheroid, while only dead cells incorporate PI and emit red fluorescence. The Image J (v 1.53K) software was used to measure the intensity of both signals for each image and the ratio of red/green emission was calculated to estimate the integrity of the spheroids. At least images of 8 spheroids were considered for each experimental condition. The experiment was replicated three times. The values obtained were expressed as percentage.

### Migratory capacity of cells grown in spheroids

To evaluate the migratory capacity of the cells grown in spheroids, immediately after treatment, spheroids were transferred to a conventional 48-well plate and allowed to attach to the substrate. Phase contrast (PhC) photographs were taken at t = 0, 24 and 48 h using an inverted microscope.

Images were captured at specific time points after attachment to calculate the total area covered by the cells forming and migrating from the spheroid, using the Image J (v 1.53K) software. The plots represent each biological replicate and condition and the mean of three biological replicates.

### PpIX production

For determination of PpIX localization, cells were seeded on 12-well plates with coverslips. After growing for 24 h and reaching 20-30% confluence, cells were treated with 50 nM SRL for 48 h. The corresponding non-treated control cells were cultured in parallel using the same experimental conditions. Following SRL treatment, cells were further incubated for 5 h with 0.5 mM MAL diluted in DMEM. Immediately after completing the incubation with MAL, cells were observed under an Olympus BX-61 epifluorescence microscope coupled to a DP70 CCD camera (Olympus) and images were captured using the CellSens Entry software. The localization of PpIX was detected as red fluorescence emission under green light excitation.

The amount of PpIX produced was also determined by flow cytometry 25. For that, cells were seeded on 6-well plates and treated with SRL; MAL; or SRL + MAL. After incubation with the compounds, cells were trypsinized with 0.1 mM EDTA/0.25% Trypsin, collected and transferred into 15 mL Falcon tubes that were centrifuged at 350 g for 10 min. The supernatant was discarded, and the pellet was suspended and fixed in 1% formaldehyde during 10 min at RT. After fixation, cells were centrifuged at 350 g for 10 min and washed with PBS (repeating these steps two times). Samples were then kept at 4 ºC until the emission was measured by flow cytometry, using FC500 Cytomics 2 Beckman lasers at λ_exc_ = 625 nm and capturing λ_em_ = 670 nm.

### ROS production

To determine ROS production, cells were seeded on 12-well plates with coverslips. When cells reached 20-30% confluence, they were incubated with SRL at 50 nM during 48 h, followed by 5 h of incubation with MAL 0.5 mM diluted in DMEM without FBS. One hour before the subsequent exposition of the cells to red light, 7.5 μM of Dichloro-dihydro-fluorescein diacetate (DCFH-DA, Abcam) was added to each well. After incubation, cells were irradiated with 3 J/cm^2^ (A431) or 6 J/cm^2^ (SCC13), rapidly washed with PBS, and freshly mounted to directly observe the fluorescent emission using a fluorescence microscope under blue light excitation. Photographs were taken and the signal was quantified using Image J (v.1.53K). The plots represent the mean fluorescence of three independent replicates.

### Indirect immunofluorescence

The localization of the studied markers was performed by immunofluorescence (IF). For this purpose, cell lines grown on coverslips to a 20-30% confluence and subjected to the previously mentioned treatments: SRL, MAL or SRL + MAL. After treatment, cells were fixed in 3% formaldehyde diluted in PBS at 4°C for 30 min and washed three times (5 min) with PBS. Then, cells were permeabilized for 30 min with 0.1% Triton X-100 in PBS, blocked 1 h with 2% BSA in PBS and incubated 1 h at 37ºC with primary antibodies **(Table 1)** at recommended concentrations indicated by the suppliers. Subsequently, cells were washed in PBS three times during 5 minutes and incubated with the secondary antibodies **(Table 2)** for 45 min at 37ºC. All antibodies were diluted in 0.5% PBS/BSA. After washing, cell nuclei were then counterstained with 1 µg/mL Höechst 33258 (*Sigma*) for 5 min at room temperature and washed three times in PBS during three minutes. Finally, coverslips were mounted with Prolong medium (*Life Technologies*). For antibody signal observation at the microscope, we employed the filters of UV (360-370 nm, UG-1 filter), blue (450-490 nm, BP 490 filter) or green (570-590 nm, DM 590 filter) excitation light filters.

### Protein electrophoresis and western blot

For protein extraction, cells were seeded on 6-well-plates and treated with the corresponding treatments. Upon removing the treatment compounds, cells were maintained in standard culture conditions for an additional 4 hour period in the case of NRF2 and p62 assays, or 24 h for the rest of the analyses. Subsequently, cells were lysed using RIPA buffer (150 mM NaCl, 1% Triton X-100, 0.05% deoxycholate, 0.1% SDS, 0.1% SDS, 0.1% deoxycholate, 0.1% deoxycholate, 0.1% SDS, 1% Nonidet-40, 50 mM Tris, pH 8) (*Bioworld*), containing phosphatase inhibitors (PhosSTOP EASYpack, Roche) and protease inhibitors (CompleteULTRA Tablets Mini EDTA-free EASYpack, Roche) for 20 min at 4°C under shaking. Cell lysates were then centrifuged at 14000 rpm during 10 min. The supernatant was collected and protein concentration was determined using the bicinchoninic acid assay (BCA, Protein Assay Kit, Pierce). Subsequently, the different extracts were properly diluted in 4x Laemmli Sample Buffer (*Bio-Rad*). The electrophoresis was performed using 7.5 or 10 % agarose gels with a Miniprotean kit (*Bio-Rad*). Proteins were then transferred to polyvinylidene fluoride (PVDF) membranes (*Bio-Rad*) using a TransBlot Turbo (*Bio-Rad*). Next, membranes were blocked with 5% Non-Fat Dry Milk (NFDM) powder diluted in Tris Saline Buffer (TBS) with 0.1% Tween® 20 and then incubated with primary antibodies **(Table 2)** diluted in blocking solution (5% NFDM or 5% TBS/BSA with 0.1% Tween® 20) overnight at 4°C. Membranes were then washed with 0.1% TBS-Tween® and incubated with the corresponding secondary antibody (peroxidase-conjugated secondary antibodies (HRP-Goat anti-rabbit IgG and HRP-Goat anti-mouse IgG, *ThermoFisher*) for 2 h at room temperature. To reveal the signal, Amersham™ ECL™ Prime (*Fisher Scientific*) and Amersham™ Imager 680 visualization system was used. The bands corresponding to different proteins were processed and quantified using ImageLab program version 2.0.1 (*Bio Rad*).

### Ultra-Violet (UV) light-induced photocarcinogenesis in SKH-1 mice and *in vivo* treatments

*In vivo* experiments using mice were carried out in compliance with the guidelines in RD53/2013 (Spain) and were approved by the Ethics Committee from Consejo Superior de Investigaciones Científicas (CSIC, Madrid, Spain) and Comunidad Autónoma of Madrid (CAM, Consejería de Medio Ambiente; Register number: PROEX 165.0/20) in the frame of the project FIS-PI21/00315 supported by the Spanish Ministerio de Economía y Competitividad.

For *in vivo* assays, SKH-1 hairless mice were employed. SKH-1 mice are immunocompetent but display high susceptibility to develop skin lesions induced by chronical exposure UVR. The time required for the development of these lesions varies depending on the spectrum of the irradiation source, the frequency of exposure and the dose used.

In the present work, 45 SKH-1 mice (Charles River Laboratories, Barcelona, Spain) were used, all females, 5-7 weeks of age and 20-25 g body weight at the beginning of the experiment. Animals were maintained at the animal facility of the National Center of Biotechnology (CNB, Madrid, Spain), in a room at 21 ± 3ºC, with automatic light/dark cycles of 12 h and a relative humidity of 40-60% and received food and water *ad libitum*.

Mice were distributed in four groups: UV (N = 15), UV + SRL (N = 10), UV + PDT (N = 10), and UV + SRL + PDT (N = 10). All the animals were subjected to chronic UVR sessions, three days a week, for 117 days to develop skin tumors.

The irradiator was designed in our laboratory and it contained 6 UV fluorescent tubes (Phillips TL UV, 20 W). The spectrum of this source was collected with a Solatell Sola Scope-I radiometer (Croydon, UK), from 270 to 380 nm, with a maximum peak at 312 nm and approximately 52% UVB, 47% UVA, and 1% UVC radiation (**Figure S1**). For the exposure to UV, the light source was placed 15 cm apart from the dorsal surface of the mice. To ensure the most homogeneous exposure among mice in different cages, the irradiation order and position of the cages in the rack varied in consecutive sessions. The irradiance of the source was measured with a radiometer, adjusted to 3.1 mW/cm^2^ and monitored during the experiment once a month.

UVR was terminated when almost 50% of the mice presented a visible lesion above 8 mm^3^, at the accumulated dose of 10.53 J/cm^2^ (117 days after initiating UVR). SRL treatment was administered to the animals belonging to the groups UV + SRL and UV + SRL + PDT. SRL was dissolved at 20 mg/mL in petroleum jelly (Lachén Laboratory, Huesca, Spain) 38 and 15 mg were topically applied on the back skin and homogeneously distributed. The application was repeated 5 times a week, starting at the end of the UVR (Day 0) and lasting until one week before the endpoint (Day 35).

PDT-treated groups received two PDT cycles (14 and 27 days after UVR termination). PDT administration consisted of topical application of a thin layer of MAL (Metvix®, Galderma, Zug, Switzerland) on the back skin, followed by 3 h incubation in the dark. After the incubation, the excess of Metvix® was eliminated with absorbent cotton soaked in 0.9% NaCl. Subsequently, mice were anesthetized with 2% isoflurane using a chamber (Ecuphar, Barcelona, Spain) and then exposed to 6 J/cm^2^ of red light (636 nm) in the first PDT cycle and to 9 J/cm^2^ in the second cycle. For the irradiation, the lamp containing a LED panel light source (Aktilite®, 50 mW/cm^2^) was placed at a 5 cm distance from the dorsal surface of the mouse. Forty-two days after the end of UVR (equivalent to 150 days after initiating the experiment), mice were euthanized using a CO_2_ chamber.

The tumor size was monitored during the experiment using an automatic calliper and the tumor volume was estimated as follows: Width x Length x (the lower measurement/2). This formula was employed due to the difficulty of obtaining a reliable measurement of the depth from the superficial tumors. The criteria to consider a lesion in the study was to reach an estimated tumor volume over 8 mm^3^. In addition, the weight of the animals was also recorded to monitor health status.

The total number of lesions (NL) on the back of each animal was monitored once a week. Mean was calculated for each time point, being relativized to the total number of mice (NM): (NL/NM).

To measure the tumor burden (TB) of each experimental group, the sum of the volumes of each tumor on the back of each mouse (TB of one mouse) resulted in the TB of each group. This TB was divided by the number of mice present at that time point. After performing a ROUT statistical test (Q= 1 %), two outliers were excluded from the study due to exaggeratedly elevated TB at Day 0: one from the UV condition and the other from the UV + PDT condition.

### Microscopy observations and statistical analysis

The microscopy images were obtained using an Olympus BX-61 epifluorescence microscope coupled to a DP70 CCD camera (Olympus), with UV (360-370 nm, UG-1 filter), blue (450-490 nm, UG-1 filter) or green (570-590 nm, DM 590 filter) excitation light filters. Photoshop CS5 (Adobe Systems Inc., USA) was used for fluorescence image processing. All experiments were repeated at least three times. For *in vitro* and *in vivo* studies, all statistical analyses were performed using version 6.05 of the graphical and statistical representation program GraphPad Prism (GraphPad Software Inc, USA).

For *in vitro* studies, statistical differences were determined by one- or two-factor ANOVA for independent samples depending on the number of variables included (*: p < 0.05; **: p < 0.01; ***: p < 0.001).

For *in vivo* studies, statistical differences were calculated through one- or two-factor ANOVAs, as differences of plotted means between conditions (adding the corresponding calculated standard deviation (SD) and indicating the number of mice (N) per condition and time-point (*: p < 0.05; **: p < 0.01; ***: p < 0.001).

## Results

### Pre-treatment with Sirolimus enhances photodynamic therapy-induced damage in SCC13 and A431 2D cultures

**Figure 1:**
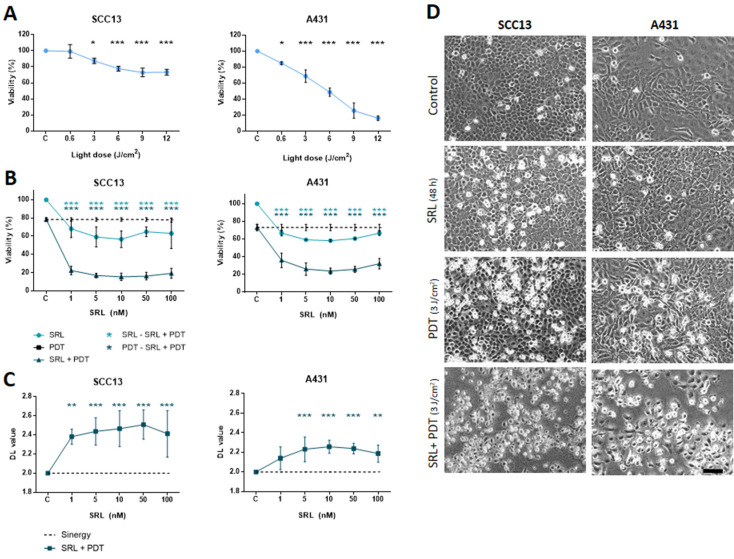
** Viability evaluation of SRL, PDT and SRL + PDT of SCC *in vitro* two-dimensional cultures. (A)** Cell survival of SCC13 and A431 24 h after PDT (0.5 mM MAL followed by variable red light doses). Cells were seeded to reach a confluence of 60-70% and 5 h incubation of 0.5 mM MAL was applied. Irradiation was performed in a range of different light doses. At 12 J/cm^2^ light dose cell viability decreases to a 70-60% viability in SCC13, whereas at about 10% of viability was detected in the case of A431 cells. * Represent statistical differences with the control group. **(B)** Cells were seeded and treated with SRL, PDT (12 J/cm^2^) or SRL+ PDT respectively. Survival of SCC13 or A431 at 24 h was measured after the treatments finalized. The results obtained showed a higher decrease in cell survival after SRL + PDT compared to that obtained after SRL or PDT alone. Cell survival was determined by the MTT assay. * Represent the differences between conditions, as described in the legend. **(C)** SRL and PDT combination displayed synergistic effect on cell viability in both, SCC13 and A431 cell lines. * Represent statistical differences with the control group. The synergy/antagonism parameter DL (difference in logarithm) was calculated as follows: *DL = (logarithm of cell survival percentage SLR + logarithm of cell survival percentage PDT) - logarithm of cell survival* percentage combination. Values are mean ± S.D. of three independent determinations. **(D)** Morphology of SCC13 and A431 cells in PhC after the treatments. Cells treated with 48 h SRL with LD20 PDT (0.5 mM MAL followed by 3 J/cm^2^ red light irradiation) or the combination of SRL + PDT were observed 24 h after later. Note the increasing number of cells with clear rounding morphology, particularly after the combined treatment. Scale bar: 100 μm. (* p < 0.05; ** p < 0.01; *** p < 0.001) (n = 3).

To study the impact of SRL treatment prior to PDT with MAL on cell survival, we first evaluated the effects of both treatments independently in SCC13 and A431 cells grown in monolayer **(Figure 1)**. Treatment with SRL at varying concentrations (1 to 100 nM) for 48 h resulted in a slight decrease in viability in both cell lines, measured by the MTT assay **(Figure S2)**. For PDT, cells were treated with 0.5 mM MAL for 5 h, according to previous results obtained in our laboratory 41, 42, and then subjected to red light. The results obtained indicated a dose-dependent response (0.6 to 12 J/cm^2^). Cell viability rates after PDT were lower in A431 compared to SCC13 cells in response to gradually increasing doses of MAL-PDT. In SCC13, a Lethal Dose (LD) of 20-30% corresponded to a dose of 6 J/cm^2^, whereas in the case of A431 3 J/cm^2^ were sufficient to induce similar values of lethality** (Figure 1A).**

For the combination assays, we treated cells in parallel with different concentrations of SRL (light blue line) or PDT alone (dotted line, corresponding to the PDT dose to induce 20-30% lethality), for the sake of comparison. The results revealed that treatment with SRL prior to PDT (dark blue line) induced a higher significant decrease in cell viability (around 70-80% lethality) compared to the individual treatments, independently of the SRL concentration used (5 to 100 nM) **(Figure 1B)**.

The interaction of the effect of both treatments was estimated as difference in logarithms (DL) and calculated as follows: DL = (logarithm of cell survival percentage SLR + logarithm of cell survival percentage PDT) - logarithm cell survival percentage combination. A value significantly lower than 2 means there is an antagonistic effect. A value similar to 2 reveals that the effects of both therapies are additive and a value higher than 2 shows the presence of a synergistic effect. The values of the parameter DL (difference in logarithm) were significantly higher than 2 in both cell lines, confirming a synergistic effect of pre-treatment with SRL before MAL-PDT **(Figure 1C)**.

PhC images of SCC13 and A431 cells after the treatments were captured to study cell morphology. The results indicated that a 48 h SRL treatment did not induce substantial cell morphological changes. However, retracted cells with a rounded morphology were observed 24 h after PDT. This morphology was more evident in both cell types after the combined treatment **(Figure 1D)**.

Cell death induced by the treatments was verified by indirect immunofluorescence for cytochrome C (Cyto C) and active caspase 3 (Casp 3). Untreated cells showed an intense fluorescence in the mitochondria net due to Cyto C localization. However, 24 h after the combined treatment, relocation of Cyto C could be observed as a diffuse green fluorescence signal in the cytoplasm in both cell lines compared to the rest of the conditions **(Figure S3A).** The indicated changes were accompanied by alterations in the morphology of the nuclei. Chromatin aggregations, typical of cell death, were revealed by H-33258 DNA staining. In SRL + PDT treated cells, we also found higher levels of nuclear signal due to Casp 3 activation (around 70/80 %) compared to PDT alone (around 20 %). No expression of active Casp 3 was observed in treated cultures with only SRL **(Figure S3B & C)**.

### SRL + PDT combination provokes damage in cutaneous SCC three-dimensional models

**Figure 2:**
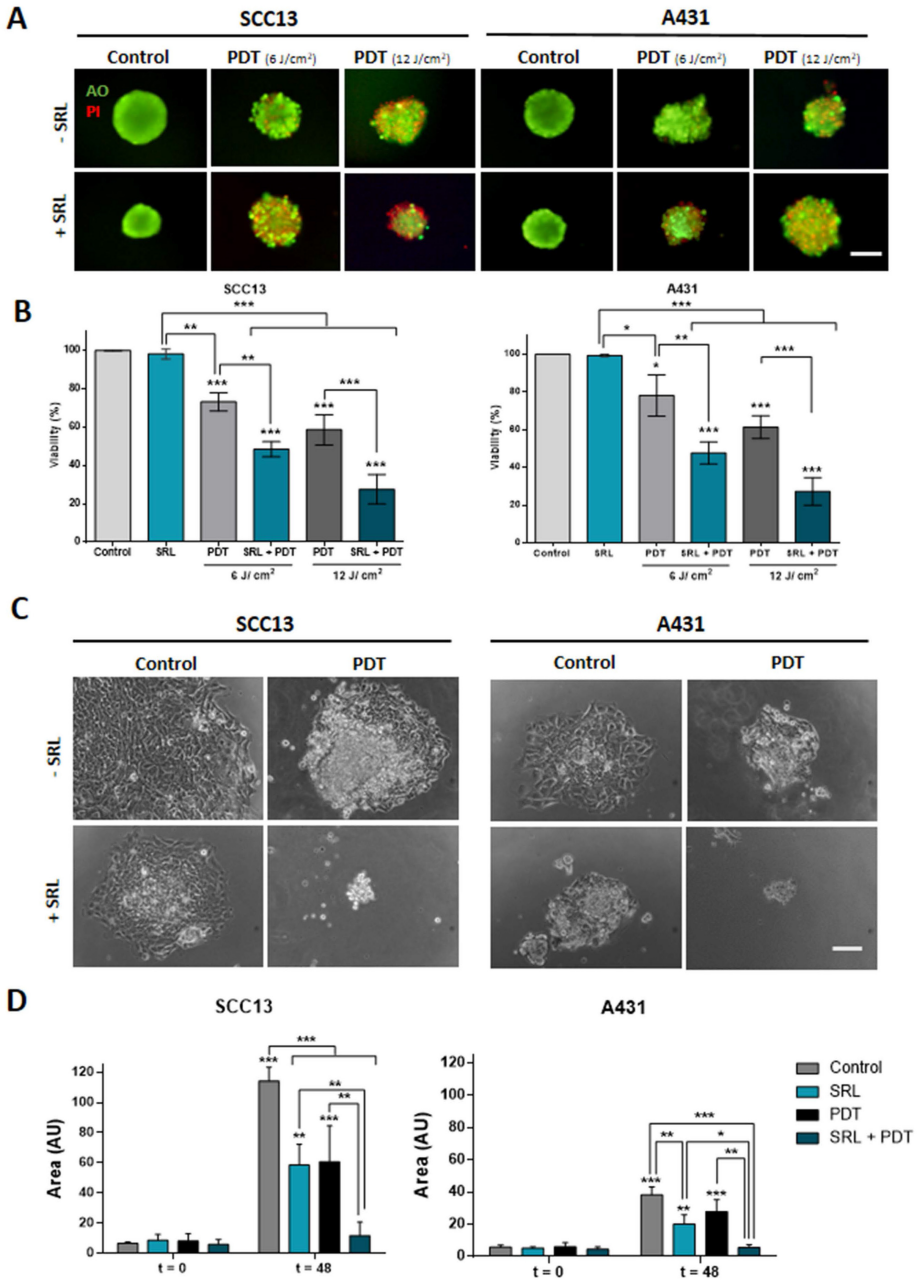
** Viability evaluation and migration of SCC *in vitro* three-dimensional models subjected to SRL, PDT and SRL + PDT. (A)** Representative fluorescence microscopy images of SCC13 and A431 spheroids formed seeding cells in non-attaching conditions and treated with 50 nM SRL, PDT or SRL + PDT (0.5 mM MAL, 6.0 and 12.0 J/cm^2^ red light irradiation doses), 24 h after irradiation, spheroids were evaluated through the AO/PI assay. All cells were visualized in green under blue light irradiation (450-490 nm); and dead cells were fluorescing in red under green light irradiation (570-590 nm). Scale bar: 100 µm.** (B)** Quantification of cell survival through AO/PI staining in SCC13 and A431 spheroids in response to the treatments employing Image J (v 1.53K). Spheroids after SRL + PDT presented a significant lower viability compared to those treated with PDT or SRL individually. **(C)** Representative PhC microscopy images of SCC13 and A431 spheroid placed in an adherent 48-well plate that disseminate 48 h after treatments (SRL, 0.5 mM MAL and 3 J/cm^2^ red light). 3 J/cm2 were employed as fluence dose due to lack of attaching at 6.0 and 12.0 J/cm^2^ red light. Only spheroids exposed to SRL + PDT treatment were not able to attach to the plate and disseminate. Scale bar: 100 µm** (D)** Quantification of SCC13 (left panel) and A431 (right panel) spheroid dissemination in response to the treatments. Quantification of disseminated area was done from three independent biological replicates from at least four different spheroids per condition. * Above each condition represents the differences between t = 0 h and t = 48 h for each condition. Differences between groups have been indicated with asterisks over horizontal brackets. Error bars denote + S.D. (*: p < 0.05; **: p < 0.01; ***: p < 0.001) (n = 3).

To assess the combined effect of SRL + PDT in three-dimensionally cultured cells, spheroids of SCC13 and A431 cells were formed and treated as in the case of monolayer cultures. The treatment conditions used were 50 nM SRL for 48 h and PDT light doses of 6 and 12 J/cm^2^ in A431 and SCC13 respectively, which were selected considering that spheroids are more resistant to PDT than monolayer cultures. Cell viability of spheroids was evaluated using the AO/PI assay (AO emitting in green, and the PI emitting in red). The results were in agreement with those obtained in the cells grown in monolayer. Furthermore, a decrease in cell viability in spheroids was detected after PDT specifically in response to 12 J/cm^2^. The PI signal revealing cell death was higher upon SRL + PDT treatment, compared to single treatments with SRL or PDT in both SCC cell lines. Quantification analysis confirmed that the combined SRL + PDT treatment results in greater cell death than the individual treatments** (Figure 2A & 2B)**. Subsequently, we evaluated the migratory capacity of cells grown in spheroids and attached to culture plates after the treatments. Spheroids treated with SRL, PDT or SRL + PDT with a dose of 12 J/cm^2^ failed to attach to the plate. However, the results obtained with a lower light dose of 3 J/cm^2^ indicated that spheroids of both cell lines, seeded just after treatment (t = 0) with SRL or PDT alone, were able to attach to the plate and cells migrated away. The total area covered by the attached spheroid and the cells migrating was significantly increased 48 h after such treatments **(Figure 2C & D)**. In contrast, in the case of the SRL + PDT combination, the majority of SCC13 and A431 spheroids were unable to attach to the plate and spread, with the covered area being significantly lower than spheroids treated with SRL or PDT alone **(Figure 2D)**.

### SRL partially re-sensitizes PDT resistant SCC13 and A431 cell lines

Taking into account that SRL was able to increase the response to PDT of both cell types in 2D and 3D models, we evaluated the ability of this compound to sensitize resistant lines to PDT. The resistant SCC13 and A431 cells, SCC13 10G and A431 10G were established by subjecting original cells to 10 cycles of PDT in ascendant doses 25, 34. SCC13 and A431 were subjected to 10 consecutive MAL-PDT cycles to obtain resistant cells. To perform this procedure, the concentration of MAL was set at 1 mM (5 h of incubation) and the red light dose was gradually increased in successive cycles to induce a survival rate lower than 20%.

**Figure 3:**
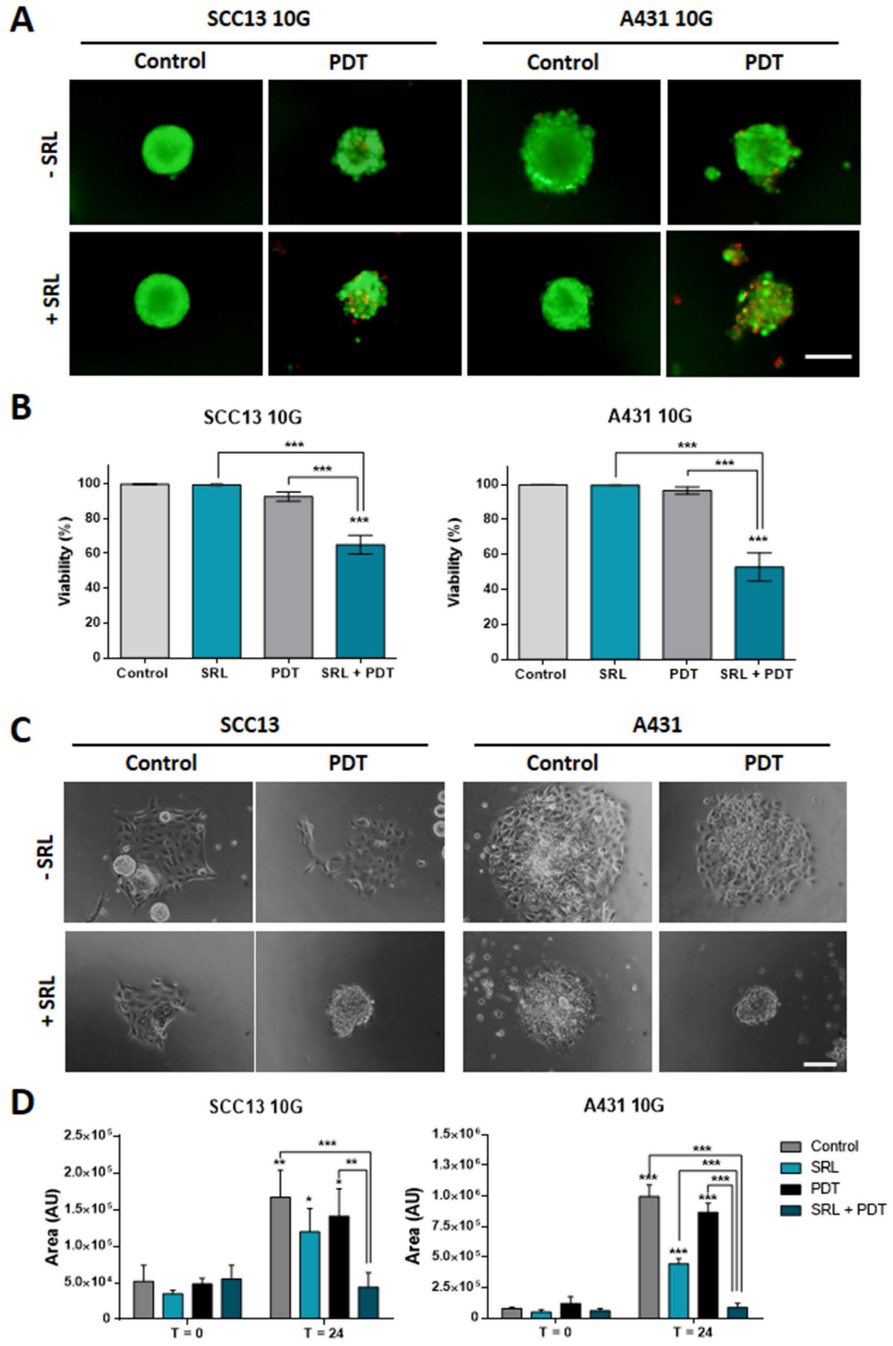
** 10G PDT resistant cells of SCC13 and A431 treated with SRL, PDT and SRL + PDT on three-dimensional models. (A)** Representative fluorescence microscopy images of SCC13 10G and A431 10G formed spheroids from seeding cells in non-attaching conditions and treated with 50 nM SRL, PDT or SRL + PDT (0.5 mM MAL and 12.0 J/cm^2^ red light irradiation doses), 24 h after irradiation, spheroids were evaluated employing the AO/PI assay. SRL + PDT treatment displays a higher PI signal PDT alone. Images are represented as merge of PI and AO photographs. All cells are visualized in green under blue light irradiation (450-490 nm) and dead cells in red under green light irradiation (570-590 nm). Scale bar: 100 µm. **(B)** SCC13 10G and A431 10G spheroids viability quantification in response to SRL, PDT or SRL + PDT) evaluated by AO/PI assay. **(C)** SCC13 10G and A431 10G spheroids were placed into an adherent 48-well plate and disseminated 24 h after treatments, photographs were taken 24 hours after. SRL and PDT treated cells could disseminate, however SRL + PDT spheroids were not able to attach to the plate and cells disseminate. Scale bar: 100 µm.** (D)** Quantification of SCC13 and A431 10G spheroid dissemination in response to the treatments. * Above each condition represents the difference between t = 0 h and t = 24 h for each condition. Differences between groups have been indicated with asterisks over horizontal brackets. Error bars denote + S.D. (*: p < 0.05; **: p < 0.01; ***: p < 0.001) (n = 3).

As the resistant cell lines were less susceptible to PDT than the parental ones, the red light dose used for the experiments with resistant cells was increased to 12 J/cm^2^. The results obtained indicated that spheroids did not exhibit a significant PI signal after PDT, as expected **(Figure 3A)**. However, PI signal was significantly higher when SRL was applied prior to PDT indicating that the combination induced a significant damage to spheroids formed by PDT resistant lines, compared to any other condition (PDT or SRL alone) **(Figure 3B)**. In addition, since PDT-resistant lines develop more aggressive features 16, we also evaluated their migratory capacity after treatments. As shown in the images, 10G resistant cells grown in spheroids were able to migrate after PDT (12 J/cm^2^). However, spheroid cells did not show migratory capacity upon treatment with SRL + PDT **(Figure 3C)**. The area covered by spheroid's cells 24 h after SRL + PDT treatment was reduced compared to those subjected to single treatments with SRL or PDT alone **(Figure 3D)**.

### SRL + PDT lower the number of lesions and decreases the growth rate in immunocompetent mice models

**Figure 4:**
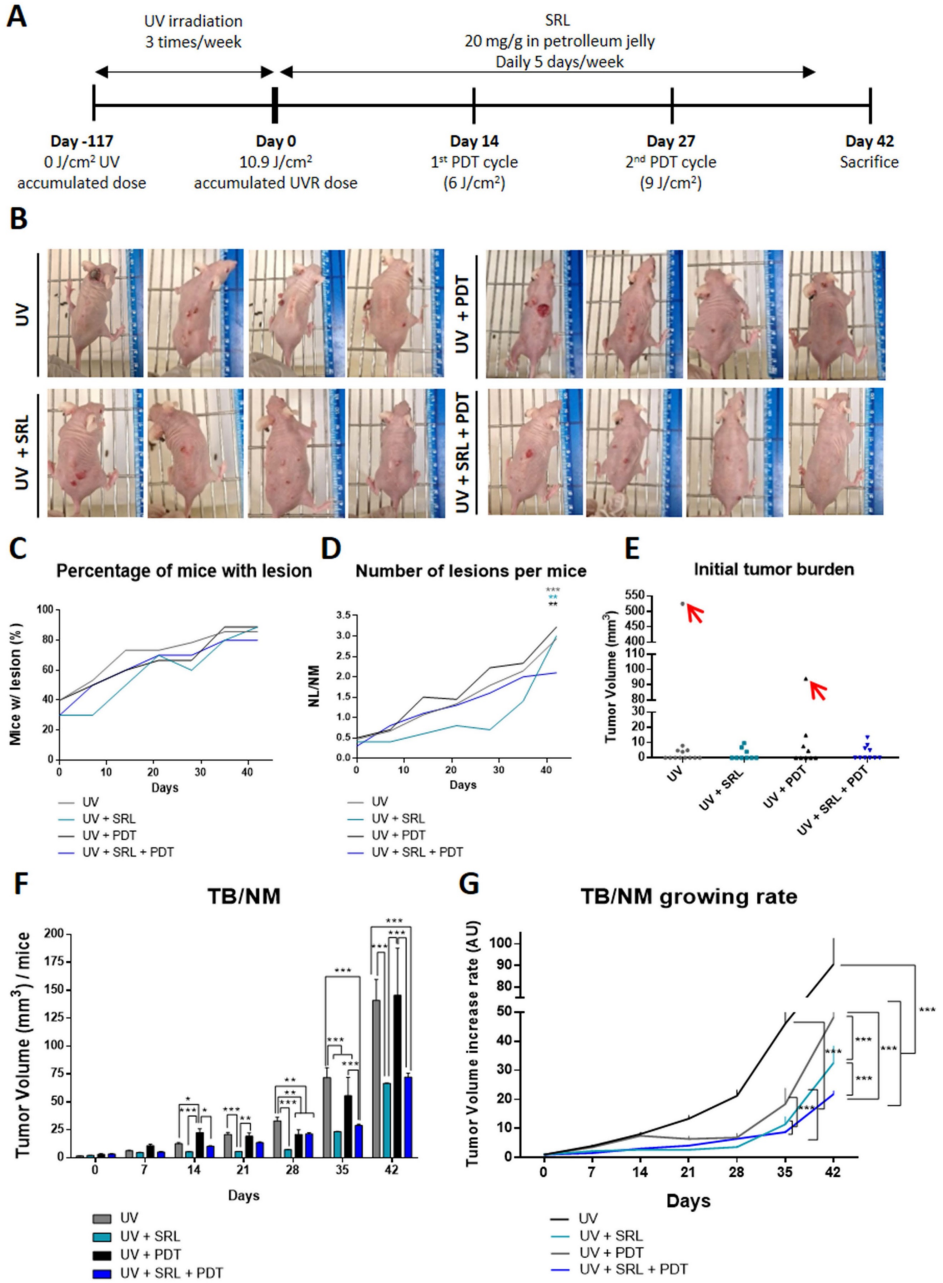
** Evaluation of SRL + PDT treatment in SKH-1 mice subjected to UV exposure. (A)** Representative scheme of timeline of the animal experiment protocol. Mice were irradiated during 117 days three times a week. Day 0 indicates the moment that UV irradiation stops (117 days after initiating the experiment) and SRL treatment (20 mg/mL) is initiated. PDT was applied 14 and 27 days after the finalization of the UV irradiation. Endpoint of the experiment was 42 days after UVR finalisation. **(B)** Images of four representative mice per group of for different conditions.** (C)** Percentage of mice with visible lesions. All four conditions reached between 70 and 90 % tumors with time after finishing UV irradiation. **(D)** Total number of lesion (NL) per Number of mice (NM) ratio; this ratio was lower in the mice group subjected to UV + SRL + PDT. * Above each condition represents the difference compared with the initial point. The color indicated the condition * is referring to and the order from top to bottom is conserved. **(E)** Initial total TB of each mouse**.** Two mice were excluded as outliers using the ROUT test (Q= 1 %).** (F)** Tumor volume per mice was calculated measuring the Total burden (TB) per number of mice (TB/NM) ratio. At day 42, SRL treated groups presented significantly lower TB/NM ratio than UV and UV + PDT groups. * Above each condition represents the difference compared with the control condition. Differences between groups have been indicated with asterisks over horizontal brackets. **(G)** Increase of TB/NM relativized at 0 day. At day 42, UV displayed higher increase in TB that the rest of conditions. SRL present the minimal increasing of tumor volume (*: p < 0.05; **: p < 0.01; ***: p < 0.001).

Given the excellent results obtained *in vitro* (both in monolayer and in spheroids) in response to pre-treatment with SRL prior to PDT, we decided to move onto *in vivo* systems to evaluate such combined treatment. For that, we have used a photocarcinogenesis development assay using SKH-1 mice chronically exposed to UVR **(Figure 4A)**. In this *in vivo* model, mice were irradiated for a total of 117 days, then they were treated with 20 mg/g topical SRL (listed as Day 0) (5 days a week for 42 days). We also applied two sessions with MAL-PDT using doses of 6/cm^2^ and 9 J/cm^2^ at 14 and 27 days, respectively, after starting treatment with SRL. On day 42 after completing the UVR exposure protocol (159 days after initiating the experiment), mice were sacrificed. In **Figure 4B,** representative photographs to illustrate skin lesions developed by mice after the different treatments are shown. As shown in the images, mice of the UV + SRL + PDT group have a reduced number of lesions. SRL treated mice seemed to present lower tumor sizes than UV and UV + PDT. PDT-treated groups presented little injuries on the skin of some mice, a product of skin scratching (**Annex 1**). At 42 days after UVR exposure, 80-95% of mice had developed visible lesions (volume> 8 mm^3^) (**Figure 4C**). Additionally, the treatment with SRL + PDT resulted in a lower number of lesions relativized to the number of mice (NL/NM), reaching values around 2 lesions per animal, not significant compared to the initial NL/NM. In contrast, the mice in the other conditions displayed a higher ratio (around 3 lesions per mouse), being significantly higher than its initial NL/NM at Day 0. The NL/NM was stabilized in SRL + PDT-treated mice in the last week, whereas SRL-treated mice was significantly increased once the treatment was removed **(Figure 4D)**. The body weight was preserved in all groups along the experiment **(Figure S4)**.

In relation to tumor burden (TB) measurements, we first analysed initial TB and excluded outliers from the study: one mouse from the UV group and another one from UV + PDT condition, marked in red in **Annex 1**, due to excessive initial TB at Day 0 **(Figure 4E)**. Then, the TB/NM ratio was calculated with time after initiating the SRL treatment. UV + PDT group presented a significant lower TB/NM ratio than that of UV from day 21 to day 35. However, after day 35 no significant differences with the UV irradiated group were found. In contrast, the UV + SRL + PDT condition presented a significantly lower TB/NM ratio than the UV and UV + PDT groups from day 28 onwards. No significant differences in TB/TNM between UV + SRL and UV + SRL + PDT were found at the end of the experiment (day 42 after initiating SRL treatment) **(Figure 4F)**. Due to no differences in the final TB/NM when comparing both SRL-treated groups, we wanted to check the tumor growth ratio of the different groups from the beginning of the treatments until the end of the experiment.

For that, we considered the TB per mice at Day 0 (Initial tumor burden), and compared it to the TB per mice at different time point until Day 42 (Final Tumor Burden). The normalization of the TB to its initial value allows plotting a curve of the time course of TB/NM growing rate. The results revealed that every treated group presented a significantly lower increase in TB/NM than the UV alone condition for day 35 to day 42 (endpoint), with no significant differences between them at day 35. The differences were observed at day 42. The UV group TB/NM growing rate is elevated and significantly higher than the treated conditions. At the same time, the TB/NM growing rate of UV + PDT group was significantly higher than SRL-treated groups. Between these ones, the condition of UV + SRL + PDT presented the lowest TB/NM growing rate, being significantly lower when compared to UV + SRL condition **(Figure 4G).**

### Combination of SRL + PDT increases PpIX and ROS levels in two cutaneous SCC cell lines

Trying to uncover the molecular mechanisms subjacent to the results obtained *in vitro* and *in vivo* in response to the combination of SRL + PDT, we evaluated different molecular pathways involved in PDT efficacy, starting with the evaluation of the potential relationship between PpIX and ROS production in monolayer cultures of SCC cells. The selected treatment conditions were the same as those chosen for the combined treatment: 50 nM SRL pre-treatment followed by 5 h of 0.5 mM MAL incubation and 3 J/cm^2^ for A431 and 6 J/cm^2^ for SCC13.

**Figure 5:**
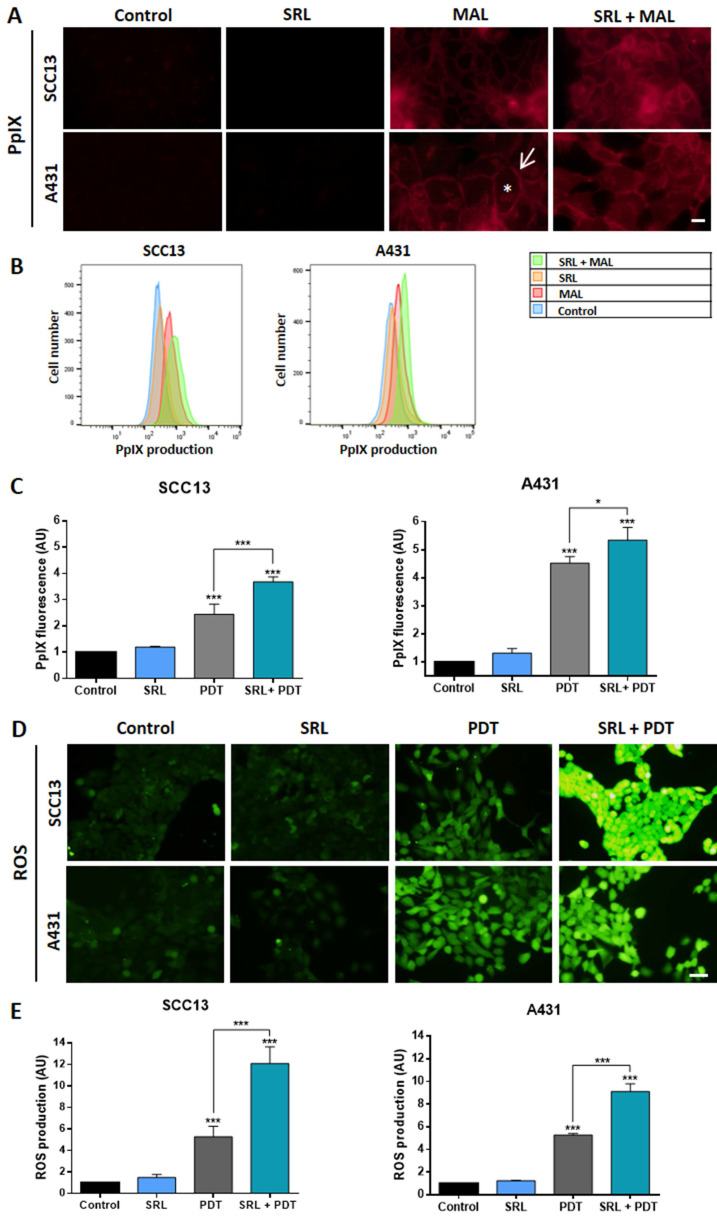
** PpIX and ROS evaluation of *in vitro* SCC13 and A431 monolayer cultures (A)** PpIX localization in SCC13 and A431 cells evaluated after 5 h of incubation with its precursor MAL (0.5 mM) and previously treated or not with SRL (48 h) on cells seeded over coverslips. Representative images were obtained by fluorescence microscopy under green light irradiation (570-590 nm). The asterisk in white represent cytoplasmic signal and the arrow represents membrane signal. Scale bar: 10 µm. **(B)** Representative histogram overlap from representative data obtained by flow cytometry for SCC13 and A431. **(C)** Quantification of PpIX production from the data obtained by flow cytometry from three different experiments. **(D)** ROS production observed in the fluorescence microscopy under blue light irradiation (450-490 nm). Cells were seeded on coverslips and treated with SRL, PDT or SRL + PDT. 1 hour before ROS detection, DCFDA sensor was administered at a concentration of 7.5 μM to detect ROS. Images were taken immediately after PDT, previously or not treated with SRL. Scale bar: 50 µm. **(E)** Quantification of ROS fluorescence after the different treatments. * Above each condition represents the difference compared with the control condition. Differences between groups have been indicated with asterisks over horizontal brackets. (*: p < 0.05; ***: p < 0.001) (n = 3).

Under the fluorescence microscope, we have observed that after incubation with MAL, PpIX signal was detected at the cell membrane and diffusely in the cytoplasm, in both cell lines. This PpIX location was not altered by SRL treatment that seems to increase the fluorescence signal **(Figure 5A)**. PpIX levels were quantified by flow cytometry, confirming that pre-treatment with 50 nM SRL before incubation with MAL significantly increased PpIX levels in both SCC cell lines **(Figure 5B & C)**. Subsequently, we evaluated ROS production using the sensor DCFH-DA, following the same previously established conditions. PDT treatment significantly increased ROS levels in SCC cell lines when compared to their control (untreated) counterparts. This increase in ROS production was even higher in cells subjected to SRL and PDT treatment with SRL + PDT, as observed under fluorescence microscopy **(Figure 5D)**. DCFH-DA fluorescence was quantified, confirming that the combined treatments produced higher amounts of ROS than PDT alone **(Figure 5E)**.

### SRL disrupts PDT-induced p62/NRF2 pathway

Given the elevated PpIX and ROS levels induced by the pre-treatment with SRL before PDT, we analysed if the mTOR inhibitor could interfere with antioxidant response pathways. One of the main factors implicated in antioxidant responses, the p62 protein, is capable of activating the expression of the NRF2 transcription factor directly implicated in the transcription of AREs 43. Thus, we have evaluated the localization of NRF2 by indirect IF at 15 min, 4 h, and 24 h after the treatment using the selected conditions (50 nM SRL pre-treatment followed by 5 h of 0.5 mM MAL incubation and 3 J/cm^2^ for A431 and 6 J/cm^2^ for SCC13).

**Figure 6:**
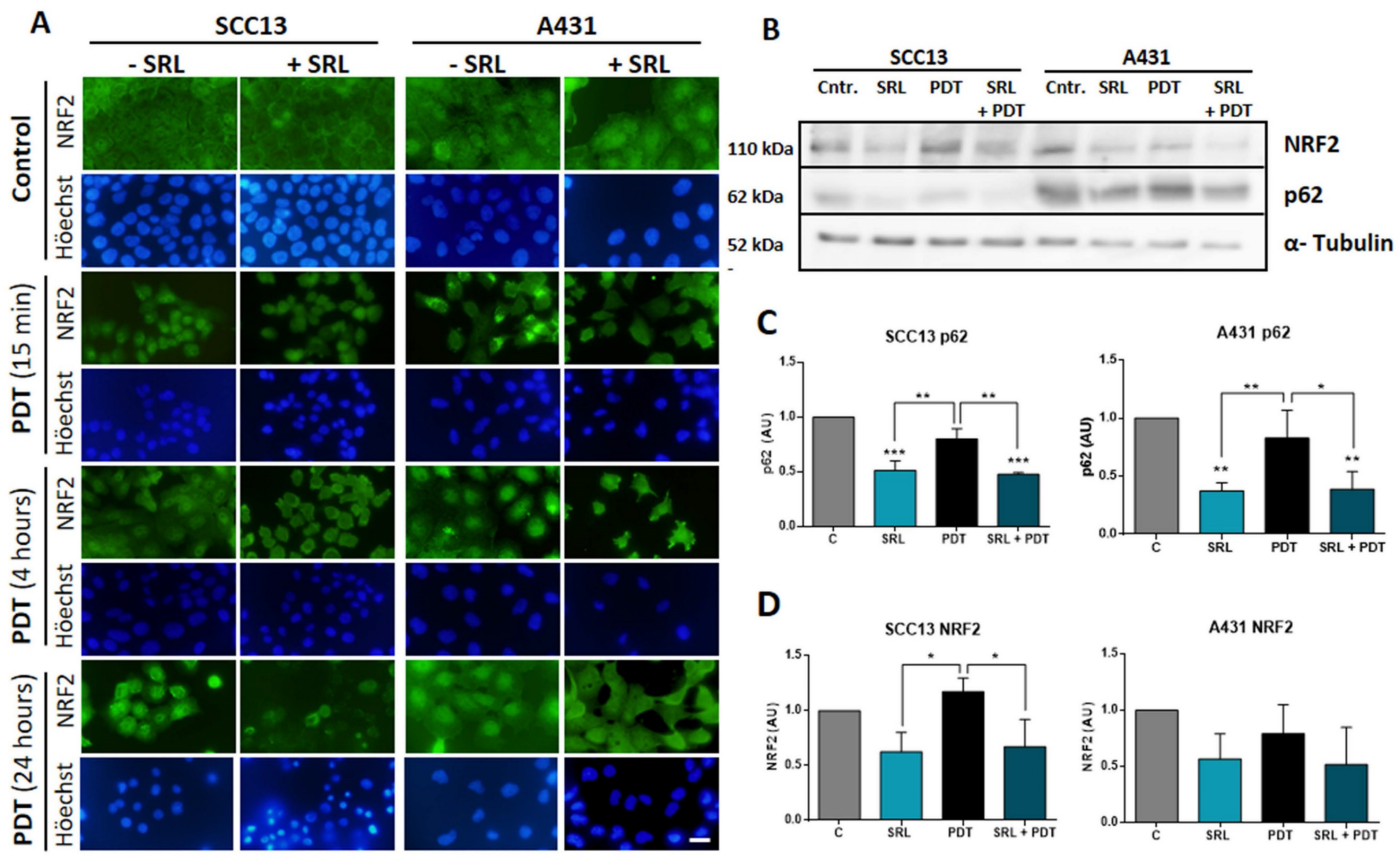
** NRF2 localization analysis and p62/NRF2 axis modulation by SRL on SCC13 and A431 cells. (A)** NRF2 localization in SCC13 and A431 cells evaluated at different time points, cells were seeded on coverslips and treated with the different conditions, fixing them at 15 min, 4 h and 24 h after PDT with MAL (0.5 mM) previously exposed or not to 48 h SRL. Indirect IF was performed to observe the localization of NRF2. Representative images were taken in fluorescence microscopy under green light irradiation (570-590 nm) nuclei were stained with H-33258 DNA staining and observed under UV light (360-370 nm). Scale bar: 10 µm. NRF2 translocation could be observed 15 minutes after PDT in SCC13 and 4 hours after PDT in A431. SRL pre-treatment before PDT prevented NRF3 relocation in both lines. **(B)** Representative images of p62/NRF2 axis modulation were obtained by WB for SCC13 and A431. WB was performed using protein extracts from three different experiments where cells were undergone to SRL, PDT and SRL + PDT for both SCC cell lines and collected 4 hours after irradiation. **(C & D)** Quantification of WB expression from the data of p62 and NRF2 images in relation to α-tubulin expression. p62 expression is decreased in SRL treated condition and NRF2 is also downregulated for SCC13. * Above each condition represents the difference compared with the control condition. Differences between groups have been indicated with asterisks over horizontal brackets. (*: p < 0.05; ***: p < 0.001) (n = 3). α-tubulin was used as loaded control.

In this sense, we have observed that untreated SCC13 cells mainly displayed cytoplasmic localization of NRF2, while it was detected at nuclear level in some cells. In the case of A431, while the majority of cells also showed cytosolic localization, greater nuclear expression was observed compared to SCC13 cells. In both lines, SRL did not cause noticeable changes in the NRF2 location. However, 15 minutes after PDT, the nuclear NRF2 was clearly seen in SCC13, while in A431 it was redistributed as perinuclear aggregates. At 4 h after PDT irradiation, NRF2 was translocated into the nucleus in both cell lines, SCC13 and A431. When SRL was applied prior to PDT, 15 min post-irradiation we did not observe NRF2 nuclear translocation in SCC13 cells nor perinuclear aggregates in A431 cells, as in the case of PDT alone. A similar trend was observed 4 h after irradiation in the cells treated with SRL+PDT, indicating SRL prevents the nuclear translocation of NRF2 in the case of both cell lines. Retracted and damaged cells were observed 24 h after PDT alone: NRF2 nuclear expression was still observed in both lines. However, when SRL pre-treatment was applied before PDT, no specific nuclear localization was detected **(Figure 6A)**.

The expression of p62 and NRF2 was also analysed by Western blot. SRL treatment induced a significant decrease in p62 in both cell lines and in NRF2 protein expression in SCC13, but this was not observed in A431. In addition, SRL treatment significantly decreased p62 expression in both cell lines **(Figure 6B, C & D)**.

### NRF2-dependent antioxidant response axis is suppressed by SRL treatment prior PDT

**Figure 7:**
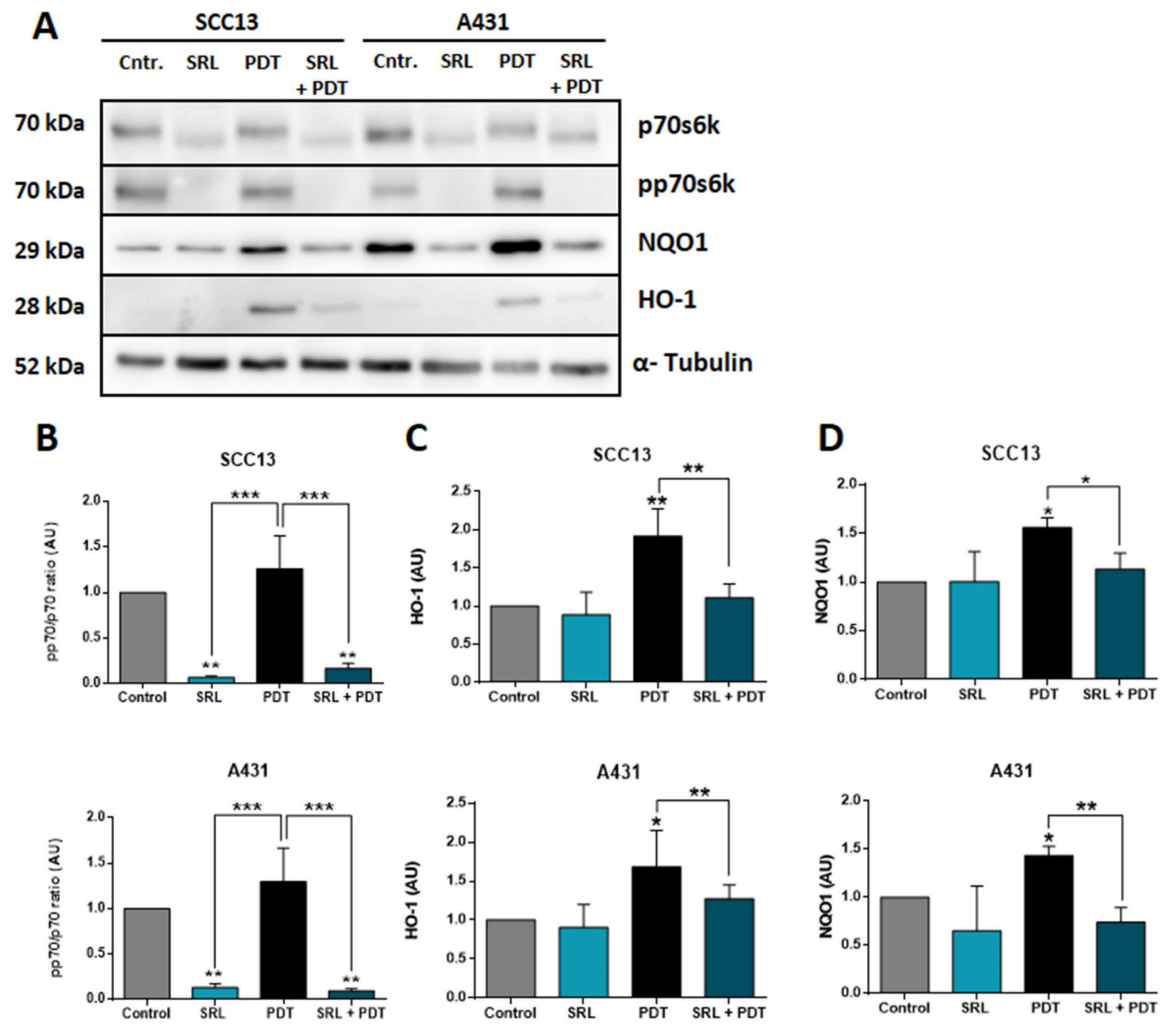
** Western Blot evaluation of the mTORC1 inhibition and AREs expression after SRL, PDT and SRL + PDT on cutaneous SCC cell lysates. (A)** WB representative images showing the expression of p70s6k, pp70s6k, NQO1 and HO-1 in SCC13 and A431 after the different treatments: 48h of SRL treatment, PDT 20/30% LD and SRL + PDT. WB assays were conducted employing protein extracts collected from three different experiments. Cells were applied SRL, PDT and SRL + PDT in both lines** (B)** pp70/p70 ratio analysis from quantification of the expression data obtained by WB. pp70/p70 ratio is significantly decreased in SRL treated cells. WB expression of **(C)** HO-1 and **(D)** NQO1 in relation to α-tubulin expression. Both molecules are induced after PDT and its levels decreased with SRL pre-treatment before PDT (*: p < 0.05; ***: p < 0.001) (n = 3)**.** α-tubulin was used as loaded control.

It is widely known that NRF2 modulates the antioxidant response against ROS by activating different genes, including HO-1 and NQO1, among others 20, 21. In this sense, we have also studied the expression of these downstream genes at protein levels by WB after treatment using the selected conditions **(Figure 7A)**. p70s6k is the immediate downstream effector of mTORC1 and its phosphorylation is dependent of the activation of this complex. SRL is a well-known mTORC1 inhibitor so the best manner to measure its effect is to measure the phosphorylation of p70s6k 26. The phosphorylation levels of p70 remained very low in SRL treated cells 24 h after treatment, indicating mTORC1 inhibition is still active **(Figure 7B)**. Evaluating AREs, downstream molecules of NRF2, in both cell lines, a significant increase in HO-1 and NQO1 expression was observed after PDT. However, SRL pre-treatment prior to PDT decreased PDT induced HO-1 and NQO1 levels, reaching values similar to those found in the control condition **(Figure 7C & D)**.

## Discussion

NMSC are the most common type of cancers worldwide, being SCC, its most aggressive subtype 1. Partial tumor remission after treatments is a relevant problem in cancer, including these types of tumors. PDT is a non-invasive therapy for treating *in situ* SCC and, in its premalignant situation, actinic keratosis. Despite the great capacity of PDT to eradicate SCC, in some cases it fails to induce a complete remission of the tumors, and therefore, relapses appear. One strategy for avoiding tumor resistance and relapses is optimizing PDT by combining this therapy with different compounds.

In this sense, our objective is the optimization of PDT by the combination of this clinical treatment with compounds that potentiate its action to destroy tumor cells and, therefore, avoid potential relapses. Many studies support the efficacy of the combination of PDT with adjuvant therapies. Studies of PDT combined with Methotrexate, Vitamin D, 5-fluorouracil and Metformin are examples of the success of PDT combinations 22, 23, 24. In fact, previous studies with indirect mTORC1 inhibitors such as Metformin revealed great capacity to potentiate PDT in SCC, both in vitro and in xenotransplants 25.

Our study proposes the use of SRL as a neo-adjuvant therapy to increase the efficacy of PDT. SRL is an immunomodulatory compound that inhibits mTORC1, approved for avoiding transplant rejection in kidney-transplanted patients. In these patients, the development of multiple SCC tumors is very common 27, 28, 29. In addition, other studies have revealed that SRL improved PDT and photothermal therapy in mice models when administered encapsulated with chlorin e6 for the treatment of metastatic breast cancer 33. Our study represents the first report to show that the combined treatment of SRL and PDT is highly effective in destroying SCC, both *in vitro* and *in vivo.* The exposure to 50 nM SRL for 48 h followed by 0.5 mM MAL incubation for 5 h and 3 J/cm^2^ for A431 and 6 J/cm^2^ for SCC13 did potentiate oxidative stress and promote tumor cell destruction. These observations were reproduced in two different types of SCC models with different levels of PDT resistance. SCC13 presented higher PDT resistance compared to A431. Among the different factors explaining the differential resistance to PDT, the capacity of the original carcinoma to resist the treatment can be highlighted. The basal expression patterns of the different cell models could also contribute to the resistance capacity. In this sense, Gallego-Rentero *et al.* 41 reported that variations in the basal HO-1 expression specifically between these two cells lines the response to PDT of SCC13 and A431.

In the same direction, spheroids constitute a more complex tumor model, bringing results closer to physiological reality 44. PDT treated spheroids of both lines presented a similar decrease in viability after the therapy. However, this result was not observed in 2D, where A431 displayed higher sensitivity than SCC13. The variability of the results between two-dimensional and three dimensional cell cultures may come from different reasons, as variations on molecular expression patterns when cells are organized in three-dimensional structures. One possible explanation could rely on the proliferation rate of A431 cells in 3D models, where they seem to proliferate less than SCC13. It is well-characterized that lower proliferation indexes seemed to be related to resistance to PDT, as other authors have demonstrated before, being related in several occasions with the epithelial mesenchymal transition, phenomenon which has also been related to therapy resistance 15, 45

Our results indicated that single treatment with SRL or MAL-PDT induced limited damage to SCC13 and A431 cells both in 2D and in spheroids. SRL reduced cell proliferation in consistence with the mTORC1 inhibition and its metabolic effects 26. The differential damage induced by PDT relies on different light doses of irradiation. The combined treatment induced strong lethality in both cell lines and spheroids when a sub-lethal dose of both therapies was employed. The produced effect was revealed to be synergetic in both cell lines. When spheroids were exposed to a specific sub-lethal PDT dose, surviving cells can migrate and disseminate in the culture plate. However, spheroids previously treated with SRL prior to PDT were not able to attach to the culture plate surface, and therefore, cells were not able to migrate. These results were observed with both cell lines (A431 and SCC13 cells). Our results are in agreement with other published studies describing the ability of the mTORC1 inhibitor SRL to reverse aggressive phenotypes in cutaneous SCC and breast cancer 46, 47.

PDT-resistant cells present greater invasiveness rates, lower epithelial phenotype, and higher capacity for tumor formation 14, 15, 16, 34. We observed that spheroids formed by PDT-resistant cell lines (SCC13 10G and A431 10G) after high doses of PDT showed no damage and significant migration and dissemination, indicating their resistance to PDT. However, pre-treatment with SRL prior to PDT increased this PDT damage on spheroids, preventing the attachment and suppressing its aggressive behaviour. Other authors have demonstrated that different mTOR inhibitors could sensitize ovarian and breast chemotherapy resistant cells 48, 49. Also, Mascaraque *et al.* 25 reported that the mTORC1 inhibitor Metformin was able to partially revert PDT resistance in *in vitro* cutaneous SCC, supporting the fact that mTOR inhibitors can revert tumor resistance.

SKH-1 mice constitute a perfect immunocompetent model for the study of skin photo carcinogenesis 50, 51, 52. UVR induces the formation of AK and SCC but not BCCs or melanomas in this animal model 53, 54. Several studies have demonstrated the efficacy of combining PDT with other compounds *in vivo*, such metformin 25, Methotrexate, Vitamin D and 5-fluorouracial 22, 23, 24.

Our results indicate that the treated group presented a diminished TB after PDT sessions but ended up elevating its TB to the control levels, in agreement with the previously mentioned studies about PDT resistance and augmented aggressiveness 14, 15. The SRL + PDT treatment was able to diminish the NL/NM and the TB/NM growing rate in comparison to the treatments alone. One week before the endpoint SRL treatment was terminated. At this time point, the SRL + PDT mice group displayed stabilized number of lesions, and moderated TB growth during the following week. Meanwhile, the number of lesions and TB considerably increased to more than double in the SRL group. These results reveal that SRL + PDT combination could offer some advantages to control more advanced tumors, limiting its growth and reducing the TNL over time. SCC, as a more aggressive type of NMSC, offers different limitations regarding effective treatment. Controlling the growing rate is key in the treatment of this type of tumors, since they usually present a field of cancerization with multiple lesions on the problematic area 55. Limiting the tumor growth rate and the number of lesions could offer advantages in clinic, especially at the long term, when most of these SCC present difficulties to be controlled.

However, we must take into consideration the action that SRL as an immunosuppressant can exert on immune cells. A potent anticancer agent of our own organism is the immune system, but in a relevant part of tumorigenic situations there is an immunosuppressive environment accompanied by haematopoietic dysregulation 56. Different subtypes of myeloid cells, such as dendritic and Natural Killer cells, are underrepresented. Furthermore, several cells of the lymphoid lineage are misbalanced as well. For instance, CD8^+^ cytotoxic T cells and CD4^+^ effector T cells are normally reduced, while CD4^+^ regulatory T lymphocytes (T_regs_) are relatively increased. A reduction in B cells, excluding the subpopulation of B_reg_ cells, has also been observed 57. This immunosuppressive environment is controlled by the tumor through the release of cytokines and other soluble factors such as TNF-α, IL-6, TGF-β, and IL-10, involved in cancer progression 58.

SRL directly affects immune cells, specifically interfering with T cell proliferation. Although in tumorigenic situations the role of SRL is still very controversial, different immunosuppressive effects have been described, such as promoting the generation of T_regs_ 59 and specific proliferation of memory CD8^+^ T cells 60. Furthermore, there are several clinical trials in different types of cancer that report SRL is able to induce immune antitumor activity 61. Taking all together, we consider that investigating the specific role of SRL in the context of immune activity during the development and progression of SCC is important to better define its potential to be translated into clinical applications.

MAL is a precursor of PpIX, the active PS molecule in PDT, which is a major element of the heme group biosynthesis pathway. Different compounds are able to modulate heme synthesis metabolism in SCC models, as a result elevated levels of PpIX 23, 24 and ROS production 25. Our results indicated that PpIX localization did not change after the combination (SRL + PDT). However, SRL was able to enhance PpIX accumulation induced by MAL as well as ROS production in both SCC lines. This increase in ROS could be explained by the largest PpIX accumulation, as other studies demonstrate 62.

The main mechanism of action of PDT is through the generation of ROS that induce tumour cell death. Therefore, preventing these ROS from being degraded by antioxidant agents is paramount. NRF2 is the main antioxidant response regulator. It has been demonstrated that the expression of p62, also known as SQSTM1, promotes NRF2 translocation into the nucleus in different pre-clinical models, favouring the expression of antioxidant enzymes HO-1 and NQO1 41, 63, 64. In addition, aberrant NRF2 expression has been found in pathological conditions, appearing translocated into the nucleus even in the absence of stressors 21. It has been reported that SRL is able to inhibit p62 expression 65. Regarding the p62/NRF2/HO-1/NQO1 axis, our results indicated that nuclear translocation of NRF2 was detected in both cell lines after PDT, whereas SRL pre-treatment before PDT prevented such translocation. This phenomenon could be related to changes in p62 expression, since p62 is able to form a complex with KEAP1, a molecule that sequesters NRF2 (NRF2/KEAP-1 complex), preventing NRF2 nuclear translocation to act as a transcription factor 47. Therefore, although further studies need to be carried out, we hypothesize that when SRL is applied, these complexes will retain NRF2 in the cytoplasm, preventing its antioxidant action.

In addition, signals downstream NRF2 could modulate PDT efficacy promoting the expression of HO-1 and NQO1 to prevent oxidative stress as a response against PDT toxicity 66, 67, 68. Our study has revealed that HO-1 and NQO1 were induced after PDT, and this was prevented by SRL pre-treatment prior to PDT in both cell lines. These results are in agreement with the previous characterization of the upstream p62/NRF2 disrupted pathway, suggesting SRL pre-treatment is impairing the triggering of a proper antioxidant response after PDT through modulation of p62/NRF2/HO-1/NQO1 axis.

Despite all the advantages SRL seems to provide, these experiments represent only a preliminary view of the landscape of SRL as an enhancer of PDT. Further *in vivo* studies using immunocompromised mice are needed, since many kidney-transplanted patients may benefit from PDT for the treatment of cutaneous SCC. In addition, there is a need to deepen our understanding of the mechanisms by which SRL + PDT could be affecting the immunological profile of SRL treated immunocompetent mice.

In addition, there is a need for a much more profound characterization the molecular pathways involved in the observed synergetic effect of SRL + PDT and how it could help to optimize the efficacy of PDT in other carcinogenic environments apart from the skin. Analysing different molecular pathways that are under the influence of mTORC1 and can modulate PDT efficacy mechanisms could explain the enhancement of SRL to PDT. In this sense, based on PpIX results, different the studies 69, 70 that relate mTORC1 depletion to deficiency in iron synthesis, the heme biosynthetic pathway could be a good candidate to study these mechanisms.

In addition, SRL can modulate many pathways by interfering mTORC1 axis, between them AREs activity. We have observed a disruption of the NRF2 axis and p62 modulation, but more studies are needed to prove that this relation. Furthermore, SRL action can modulate AREs through the disruption of p38 activity, upstream activator of AREs 71. Thus, the study of this axis could also be interesting to elucidate molecular mechanisms underlying increased PDT damage after SRL administration.

To conclude, we propose SRL as an excellent candidate to potentiate PDT as it is able to increase cell damage and decrease cell dissemination *in vitro*. In addition, SRL can revert resistance of SCC to PDT *in vitro*. *In vivo* results indicated that SRL pre-treatment prior to PDT produced a stabilization of lesions in mice induced by UV light. The molecular studies performed have indicated that SRL is able to potentiate PDT through by increasing PpIX and ROS production, responsible for cell stress and, at the same time, avoiding an efficient antioxidant response by impairing the axis p62/NRF2/HO-1/NQO1 in charge of managing created ROS, provoking an increase in tumor cell death.

## Supplementary Material

Supplementary figures.

## Figures and Tables

**Table 1:** Administered light doses for PDT resistance induction to ten cycles of PDT applied in SCC13 and A431 cell lines.

Generation	1^st^	2^ nd^	3^ rd^	4^ th^	5^ th^	6^ th^	7^ th^	8^ th^	9^ th^	10^ th^
SCC-13	1	1.125	1.125	1.35	1.35	2.7	3.6	4.5	6.75	9
A-431	1	1.125	1.35	1.8	2.25	5.4	7.65	8.1	9.9	11.7

Light doses are expressed in J/cm^2^

**Table 2:** Antibodies employed for immunofluorescence and Western Blot analysis.

Name	Supplier	Origin	Application	Reference
Primary				
Cytochrome C	Invitrogen	Mouse	IF	33-8200
Caspase 3	Cell Signaling	Rabbit	IF	9664s
NRF2	Abcam	Mouse	IF/WB	Ab89443
P62/SQSTM1	Abnova	Mouse	WB	H00008878-M01
α-Tubulin	Cell Signaling	Mouse	WB	3873S
p70s6k	Cell Signaling	Rabbit	WB	2708S
pp70s6k	Cell Signaling	Rabbit	WB	9234S
NQO1	Cell Signaling		WB	3187S
HO-1	Cell Signaling	Rabbit	WB	70081S
Secondary				
Alexa Fluor® 488	Thermo Fisher	Goat anti mouse	IF	A11001
Alexa Fluor® 546	Thermo Fisher	Goat anti rabbit	IF	A11035
anti-mouse IgG-HRP conjugated	Amersham	Goat	WB	10179202
anti-rabbit IgG-HRP conjugated	Amersham	Goat	WB	11859140

## Abbreviations

NMSC: Non-melanoma Skin Cancer
BCC: Basal Cell Carcinoma
SCC: Squamous Cell Carcinoma
PDT: Photodynamic Therapy
AK: Actinic Keratosis
PS: Photosensitizer
ROS: Reactive oxygen species
^1^O_2_: Singlet oxygen
PpIX: Protoporphyrin IX
ALA: δ-Aminolevulinic acid
MAL: Methyl 5-aminolevulinate
CAFs: Cancer associated fibroblasts
SOD: Superoxide dismutase
NQO1: NAD(P)H:quinone oxidoreductase 1
HO-1: Heme-oxygenase 1
NRF2: Nuclear factor-erythroid 2 related factor 2
UVR: Ultraviolet radiation
AREs: Antioxidant Response Elements
SRL: Sirolimus
DMEM: Dulbecco's Modified Eagle's Medium
FBS: Fetal Bovine Serum
PBS: Phosphate Buffered Saline
DMSO: Dimethyl sulfoxide
AO/PI: Acridine Orange/Propidium Iodide
PhC: Phase contrast
DCFH-DA: Dichloro-dihydro-fluorescein diacetate
PVDF: polyvinylidene fluoride membranes
NFDM: Non-Fat Dry Milk
UV: Ultraviolet
NL: Number of lesions
NM: Number of mice
TB: Tumor burden
LD: Lethal dose
Cyto C: Cytochrome C
Casp 3: Caspase 3
