# Supplementary material for: The m-TORC1 inhibitor Sirolimus increases the effectiveness of Photodynamic therapy in the treatment of cutaneous Squamous Cell Carcinoma, impairing NRF2 antioxidant signaling
Source: Int J Biol Sci. 2024 Aug 6;20(11):4238–57. doi: 10.7150/ijbs.94883 (PMC11379070; doi:10.7150/ijbs.94883)

Supplementary Material

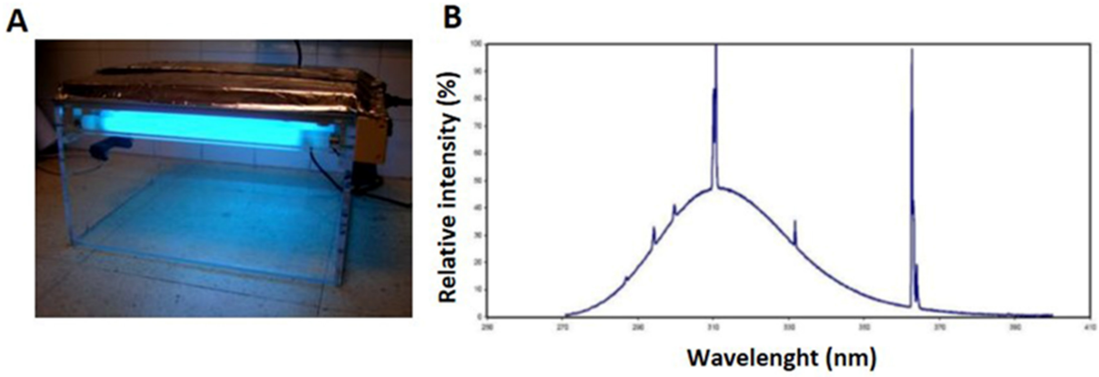

Supplementary Figure 1. UV irradiation and its absorption spectrum (A) UV light irradiator employed for UV induced carcinomas. **(B)** Emission spectrum of the UV light irradiator utilized (Phillips TL UV 20W).

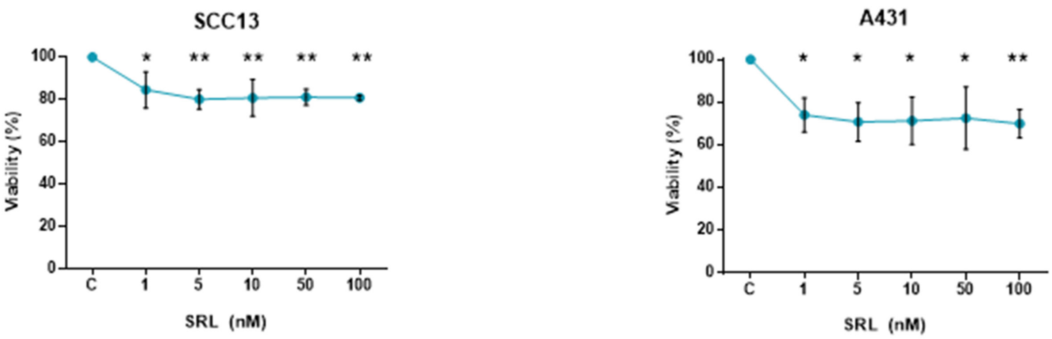

Supplementary Figure 2. Viability assessment in SCC13 and A431 cells treated with SRL. Cells were exposed to SRL at different concentrations (1 –100 nM) for 48 h. Viability was determined by the MTT assay. * Indicates significant differences compared to Control condition (*: $p < 0.05$; **: $p < 0.01$) ($n = 3$).

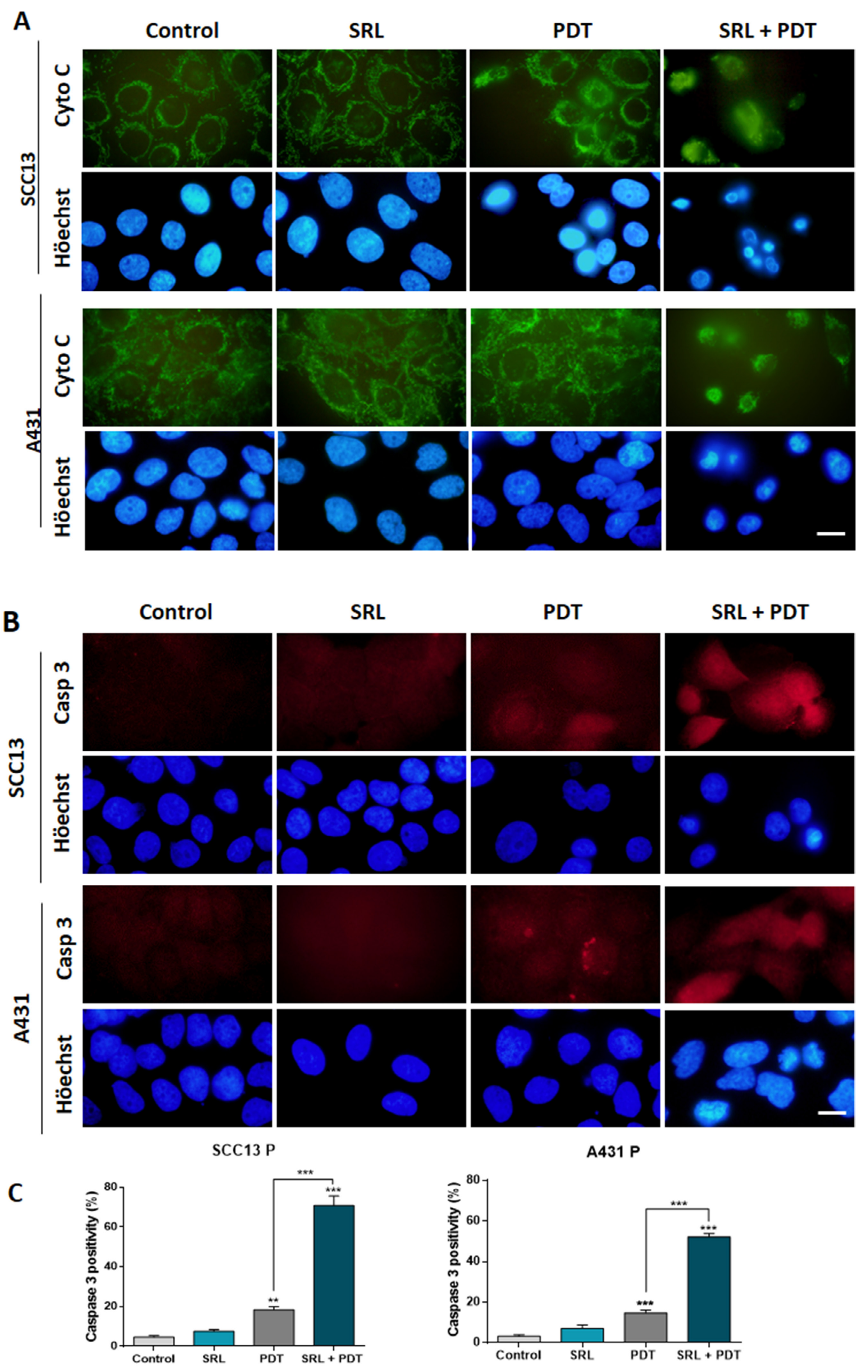

Supplementary Figure 3. Cytochrome C and active caspase 3 indirect immunofluorescence after SRL and PDT treatments in SCC13 and A431 cells. (A) Cyto C distribution fluorescing in green under blue light exciting light. Whereas in untreated and 50 nM SRL exposed cells, Cyto C is located at the mitochondrial net, it is relocated to the cytoplasm 24 h after PDT and particularly after SRL + PDT treatment. Scale bar = 10 µm. **(B)** Active Casp 3 fluorescing in red under exciting green light. Casp 3 nuclear signal is only patent after PDT and SRL + PDT. Scale bar = 10 µm. **(C)** Active casp 3 quantification from a total of 1000 cells. Values are mean ± S.D. of three independent determinations. * indicates significant differences compared to Control condition. (**: $p < 0.01$; ***: $p < 0.001$) (n = 3).

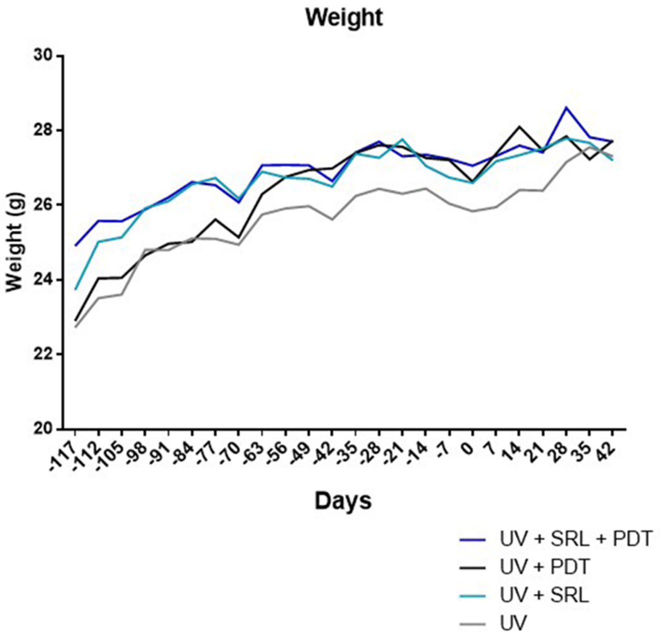

Supplementary Figure 4. Weight evolution in each group of mice. Weight is expressed as the mean per condition. Time point Day 0 corresponds to the termination of UVR chronic exposition and the start of SRL administration.

Annex 1. Photographs of all mice at Day 0 and Day 42 from the photocarcinogenesis experiment. The two mice excluded from TB studies are marked in red.

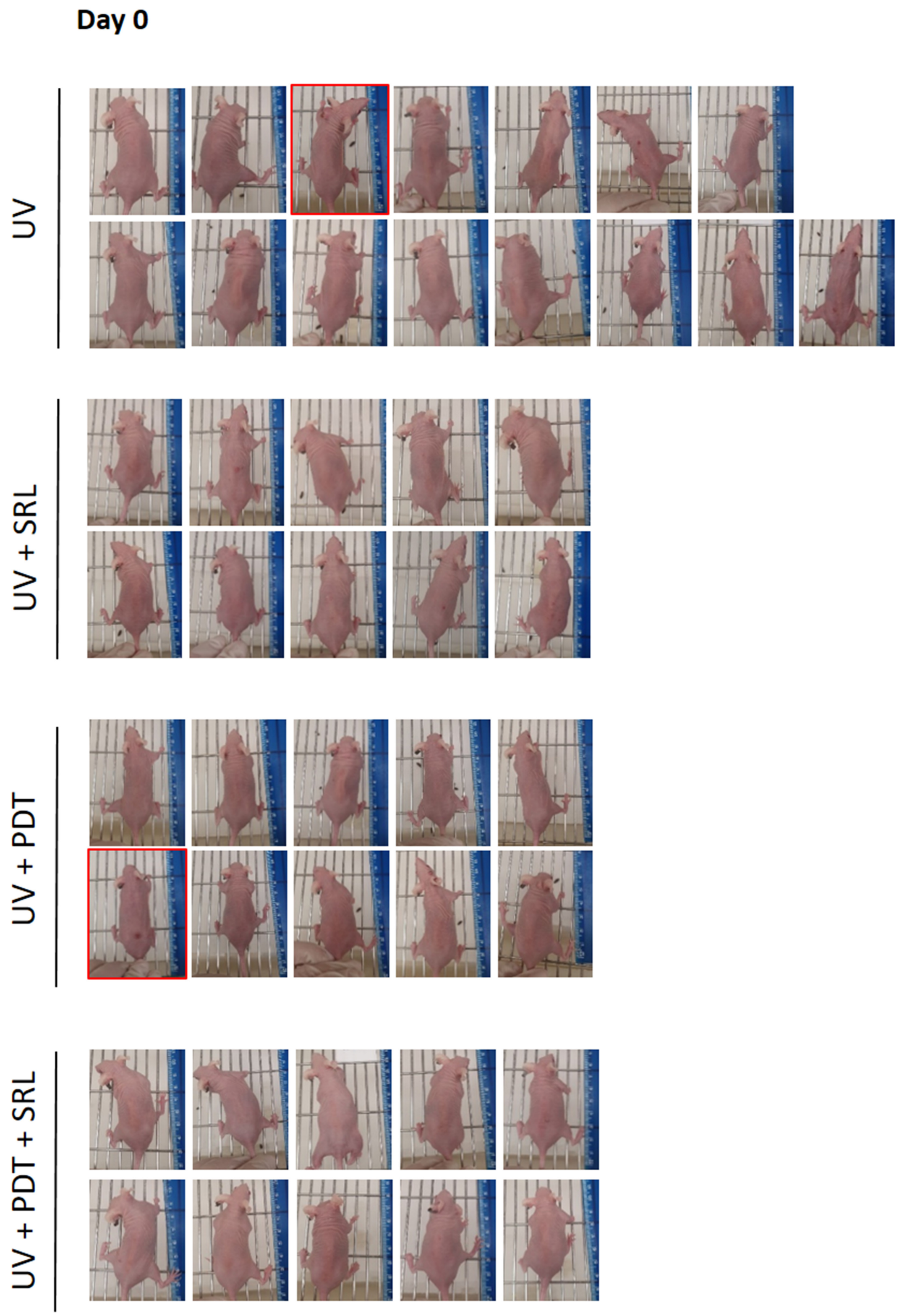

Day 42

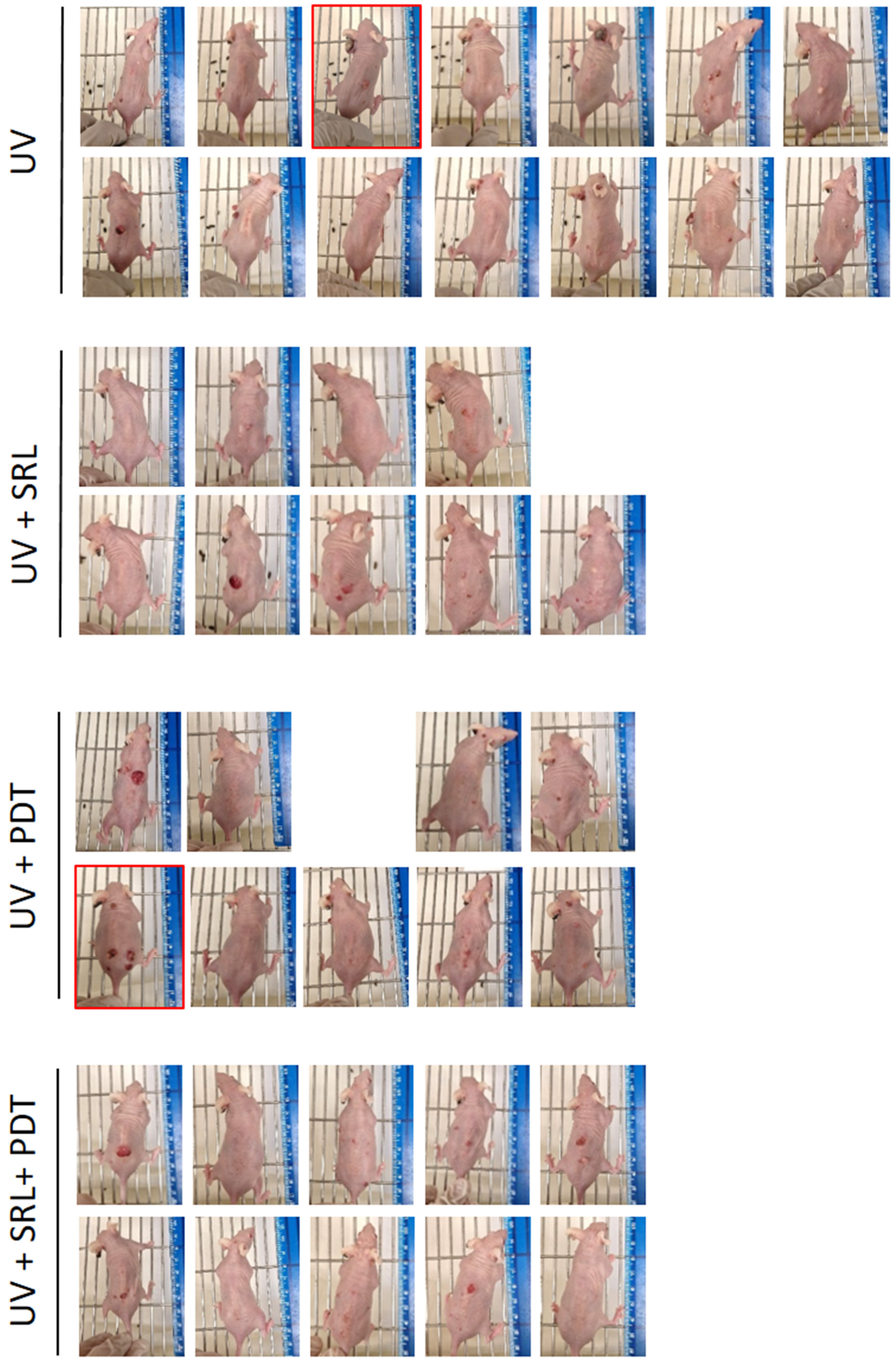

Supplement: Supplementary file 1 — Supplementary figures. [file ijbsv20p4238s1.pdf]
